# Soft Tissue Augmentation in Dermatology – 2009 Update

**DOI:** 10.4103/0974-2077.63220

**Published:** 2010

**Authors:** Michael H Gold

**Affiliations:** *Gold Skin Care Center, Tennessee Clinical Research Center, Advanced Aesthetics Medi Spa, The Laser and Rejuvenation Center, Vanderbilt University Nursing School, Nashville, TN USA; Huashan Hospital, Fudan University, Shanghai; The First Hospital of China Medical University, Shenyang, China*

**Keywords:** Dermal filler, non-invasive, hyaluronic acid, soft tissue augmentation

## Abstract

The number of products available to dermatologists for soft tissue augmentation has grown significantly over the past several years in the US. This manuscript will review the various hyaluronic acid fillers and other *Food and Drug Administration* -approved products we are utilizing for our patients in the rejuvenation process. It is hoped that through this article clinicians will feel more comfortable using these products in their everyday practice of dermatology.

## INTRODUCTION

The field of soft tissue augmentation has had a rapid growth over the past several years, mainly due to the development of the hyaluronic acid (HA) fillers and other fillers now routinely utilized for rejuvenation of the skin. These products have changed the paradigm for clinicians as we search for new ways to treat the ageing face, especially as we treat facial lines, wrinkles, and volume loss, commonly associated with facial ageing.

Over the past several years, there has been a dramatic rise in the number of ‘non-invasive’ cosmetic procedures being performed by clinicians. This trend started with the release of botulinum A toxin (Botox™), and according to the 2008 statistics from the American Society for Aesthetic Plastic Surgery (ASAPS), the injection of Botox™ continues to lead in the number of procedures being performed. It has been reported that the injection of Botox™ is the number one cosmetic procedure being performed in the world at this time. Next in line to Botox™ injections is laser hair removal, which is the second-most common noninvasive cosmetic procedure, followed closely by the injection of HA fillers, which has steadily ‘climbed’ up the ladder over the past several years. In 2008, ASAPS reported that their members utilized HA fillers in 1,262,848 procedures.[1] This trend is mirrored by other reporting organisations, including the American Society for Dermatologic Surgery (ASDS). Other fillers, not often included in these surveys, make these numbers even higher.

## IDEAL DERMAL FILLER

Clinicians have been wrestling with this concept for many years and we may just be approaching this ‘ideal’ group of products with the HA fillers and some of the other fillers now available. The fillers should be easy to inject, produce reproducible results and have a longevity profile that is satisfactory to both the patient and the physician. When we speak of longevity, most would agree that for a filler to be effective, it should last for a period of at least one year or perhaps as long as two years. Clinicians also want an ideal filler to be painless upon injection, to be non-allergenic, which means no skin testing prior to injection, non-carcinogenic, non-teratogenic, and one which, when injected, shows little migration over time. The ideal filler must have a long ‘shelf life’ and be free from all transmissible diseases. It must also have few, if any, untoward effects following the injection into the skin. Cost is also a factor for the ideal filler; the filler must be affordable to both the physician and the patient receiving it.[2]

## CLASSIFICATION

Fillers have been classified by many over the years, and it is beyond the scope of this manuscript to get into debates and discussions on the various classifications with regard to which group of fillers in these classification systems is better than the other. What is important for the discussion at hand is that there are fillers that can be classified as non-permanent and those that are considered permanent.[3] Non-permanent fillers are the most popular at this time and we continue to see an increase in their use and in the number of these fillers reaching the market. These fillers usually last upto one to two years for some products. Permanent fillers may have a role for certain patients and in the hands of skilled injectors.

In this manuscript, we will focus first on the HA fillers, a group of products which, as noted, has changed the face of soft tissue augmentation. We will also review the other Food and Drug Administration (FDA)-approved products for soft tissue augmentation, and highlight some of the current research initiatives going on in this most exciting cosmetic field.

HA fillers, as noted, is the largest group of non-permanent fillers available for soft tissue augmentation. These include Restylane and Perlane^®^, Juvederm^®^ Ultra and Juvederm^®^ Ultra Plus, Elevess (now known commercially as Hydrelle^®^), and Prevelle^®^ Silk. The newest of the collagen fillers, Evolence™, also available in the US, will be discussed, as well as, some of the semi-permanent fillers, known commercially as Radiesse^®^ and Sculptra^®^. The one permanent filler available in the US, known as ArteFill^©^, will also be described here.

## DIFFERENT FILLERS AND THEIR CHARACTERISTICS

In the US, the bovine collagen products, known as Zyderm^®^ and Zyplast^®^, were the first to be introduced to dermatologists, in the field of soft tissue augmentation in the early 1980s. It became the standard for many years and many patients experienced very impressive cosmetic enhancements as a result of the collagens. They required skin testing, and many recommended double skin testing to minimize the potential for allergy to the bovine collagen. For almost 20 years, this along with human-derived CosmoDerm^®^ and CosmoPlast^®^ were all we had; and then the doors or floodgates opened, thanks to new fillers making their way through the FDA and into the hands of dermatologists, for the benefit of our patients.[2]

### Hyaluronic acid fillers

In order to understand HA fillers, some basic terms and characteristics that make HA fillers unique, are needed. HA or hyaluronan is a glycosaminoglycan, which consists of repeating non-sulphated disaccharide units of glucuronic acid and N-acetylglucosamine.[4] HA, a naturally occurring substance, is a biopolymer and it exhibits no species or tissue specificity. HA is an essential and abundant component of the extracellular matrix in all animal tissues. HA is highly hydrophilic and therefore attracts water, and helps form large concentrations that can occupy a large volume relative to its mass. It has been found to form gels at even low concentrations. When water is drawn into the HA matrix, it creates a swelling pressure or turgor that enables the HA complex to withstand compressive forces. These characteristics and in particular, the fact that HAs do not exhibit tissue or species specificity, which plays a crucial role in minimizing any potential immunological reactions or other allergic potentials have helped make HA fillers popular among clinicians injecting patients, to improve fine lines and wrinkles and for volume enhancement.

The first HA developed as a dermal filler dates back to 1989, when Balazs *et al.* described the first injectable HA filler.[5] Although it was not a long-lasting dermal filler, the HA ‘revolution’ had begun.

### Factors which are important in characterizing a HA filler

Several differentiators have become important in their development. These include: the source of HA, the concentration of HA being utilized, the particulate size of the HA, the cross-linking of HA and the type of cross-linking agent being used, whether the HA is monophasic or biphasic, and whether an anaesthetic is added to the syringe or not. Some of the original HA fillers used avian rooster combs as the source for their HAs, but more commonly the source is bacteria-based, mainly from the fermentation of the *Streptococcus equine* bacterium. Most of the newer HA fillers have higher concentrations of HA compared to the older materials. It is felt that those HA fillers with higher concentrations of HA may be longer lasting, therefore, those with concentrations of greater than 20 mg/ml are considered ideal for HA fillers at this time. Cross-linking is important and most utilize ether cross-link bonds to help stabilize the HA. The newer non-particulate HA fillers contain double cross-linking, multiple cross-linking bonds. They may be also in monophasic gels, in an attempt to stabilize the molecule even more. This cross-linking makes the HAs less resistant to degradation, and thus enhancing longevity. As a result of the cross-linking process and non-particulate nature of newer HAs, higher HA concentrations are required to prevent biodegradation from free radicals and other enzymes. This leads to enhanced longevity of the filler. 1, 4-butandiol diglycidylether (BDDA) and 1, 2, 7, 8-diepoxyoctane are the commonly used cross linking agents. Larger HA particles tend to last longer as fillers, and are usually designed for deeper filler injections.

Both monophasic and biphasic fillers have their advantages; monophasic HA fillers are more cohesive, may last longer and may not migrate as much following its injection. However, biphasic HA fillers are more easily customized, to obtain the appropriate particle size to suit the indication and the anatomic area being treated.[2]

Numerous HA fillers are available in Europe and elsewhere around the world. In the US, due to a more stringent FDA approval process, there are fewer products available; although recently, many more are undergoing clinical evaluation through FDA-approved protocols. The remainder of this manuscript is going to focus on the fillers available in the US, reviewing their clinical studies and FDA approvals.

### Analysis of results of different fillers

a) Restylane family: The first of the ‘new’ fillers in the US was Restylane. Restylane received its FDA approval in the US in December 2003, although it received its EU clearance much earlier, in 1996. It has been injected in well over ten million treatment sessions worldwide and is considered the standard against which all other and all new HAs are measured. It is manufactured by Q-Med AB (Uppsala, Sweden) and is marketed in the US and Canada by Medicis Pharmaceutical Corporation (Scottsdale, AZ USA). Restylane is a non-animal stabilized HA, commonly referred to as NASHA, produced from the fermentation of equine streptococci. It is cross-linked with BDDA, with a 1% degree of cross-linking. Restylane has an HA concentration of 20 mg/ml and its gel particulate size is 400 μm. It has a particulate size of 100,000 gel particles per milliliter and is the first of the Restylane family of products available from Q-Med and Medicis. Restylane's FDA approval is for mid-dermal applications, such as, deep wrinkle correction, lip augmentation, nasolabial fold correction and for glabellar creases. It received its initial FDA approval for six months duration of correction. Restylane has also been successfully used in the treatment of tear trough deformities

Perlane, the second product released in the Restylane family, contains 8000 gel particles per milliliter and is indicated for deeper injections and deeper clinical defects. In other parts of the world, this product is known as Restylane Perlane.[6]

Other products in the Restylane family include Restylane Touch™, Restylane Lipp™, Restylane SubQ™ and Restylane Vital™. A newer product, known as Macrolane™, has been introduced into Europe, mainly designed for volume enhancement. Further information on these products can be obtained from Restylane website.[7]

The two pivotal European clinical trials that led to the approval of Restylane in Europe will be discussed in detail here. These trials, by Duranti *et al.*[8] and Olenius[9] showed the safety and efficacy of Restylane in the correction of the nasolabial folds. In the first trial by Duranti *et al.*, 78% of the patients who enrolled found that they were able to maintain moderate-to-marked clinical improvement for eight months following the injection. In the second study, by Olenius, there was correction of 82%, noted at 12 weeks and 69% at 26 weeks. Adverse events (AEs) noted in these two clinical trials were predominantly injection-related AEs, consisting of treatment-site erythema, hyperpigmentation at the treatment site and pain from the injection itself, reported in 13% of the patients in these trials. As experience grew with the product, injection techniques were refined, a later study of a large series of patients, Friedman *et al.*,[10] found that the injection related AE rate was, in fact, occurring in only 0.15% of the patients receiving Restylane injections.

Shortly after these reports, several cases of what was described as delayed implant hypersensitivity, were reported in European literature.[11–13] Through these evaluations, it was determined that there was a 0.4 to 3.7% risk of this occurring following Restylane implantation. As a result of this delayed implant hypersensitivity occurring in more patients than was acceptable, a more purified Restylane product was manufactured by Q-Med, and this more purified product, NASHA, is what is currently available today. Clinical evaluations with the new purified Restylane and with clinicians mastering their injection technique, AEs were reduced to 0.06% and hypersensitivity reactions were reduced to 0.02%, and threrfore considered acceptable for continued use. This helped in the acceptance of this new NASHA product on a broader basis. These factors, and the fact that HAs in general requiring no skin testing prior to injection as stated earlier, led to the commencement of the pivotal US clinical trials for Restylane.

The US clinical trials for Restylane compared Restylane in one nasolabial fold with Zyplast collagen, the standard collagen injectable material available at that time, being injected into the other nasolabial fold. In this clinical trial by Narins *et al.*,[14] 138 individuals were included for evaluation. The majority of the patients enrolled were females (93%) and Caucasians (89%). The protocol design consisted of injection in each nasolabial fold with each product for optimal correction. The patients were asked to return at two weeks for any touch-up injections if needed. Optimal correction was the goal of the injection process and patients were allowed two sessions if needed, to achieve their optimal correction. The study results showed that optimal correction was achieved in 1.4 sessions for both the products being injected. The volume needed for Restylane, for volume correction, showed a mean of 1.0 ml (range 0.3 to 2.8 ml), while the amount of Zyplast used showed a mean of 1.6 ml (range of 0.1 to 5.0 ml). The Wrinkle Severity Rating Scale (WSRS) score for Restylane was superior at all time points, as compared to the Zyplast side. This was true at two months, four months, and six months following the optimal correction of the nasolabial folds. At the six-month evaluation Restylane was rated superior in 56.9% of the patients compared to Zyplast in 9.5% of the patients. The Global Aesthetic Improvement Scale (GAIS) was also superior for Restylane at all time points, with a 62% rating for Restylane superior at six months, as compared to 8% rating for Zyplast superior.

Adverse events were evaluated at each follow-up visit during the course of the study. Mild-to-moderate injection site reactions occurred in a similar and non-statistical fashion with both the products (93.5% Restylane, 90.6% Zyplast). These were short-lasting in all cases, usually resolving within seven days. Of all treatment-related AEs during the evaluation, 26.4% were reported for Restylane and 39.1% for Zyplast. Delayed-onset reactions were noted in 8.7%; all resolved within two to three months without intervention. There were no reports of hypersensitivity reactions reported during the trial.

Further evaluations have been performed with Restylane over the past several years in the US.[15–17]The evaluations have continued to show the safety and efficacy of this product in each and every study. Two of the US clinical evaluations are very important and warrant in depth discussion. The first, by Odunze *et al.*,[18] evaluated 60 patients who received Restylane injections, one-third of whom were of darker skin types, (Fitzpatrick skin types IV - VI). They noted no untoward AEs in the darker skin color group, providing evidence that Restylane can be safely injected into patients of all skin types. The second study, by Narins *et al.*,[19] also studied Restylane, but looked at repeat injections and longevity associated with repeat injections in seventy-five patients in a multicenter evaluation. The patients were randomized to receive retreatment of one of their nasolabial folds at 4.5 months and the contralateral fold at nine months after correction of both folds at the initial visit. Results were presented and analyzed at 18 months. The WSRS improved significantly (p < 0.001) from baseline, with mean improvement noted from 1.1 to 1.7 grades. Ninety-seven percent of all the patients responded to this retreatment program, and the efficacy of the retreatment schedules did not differ significantly. The AEs reported were all local and consisted of swelling and bruising at the treatment site, which occurred in 33%, and were not rated as serious in this study. Thus, Restylane was shown to maintain correction for 18 months following a repeat injection at 4.5 months. This study led to a second submission to the FDA, known as a supplemental PDA, for its label, giving a new indication for Restylane longevity up to 18 months with a repeat injection at 4.5 months.

**Figure 1:**
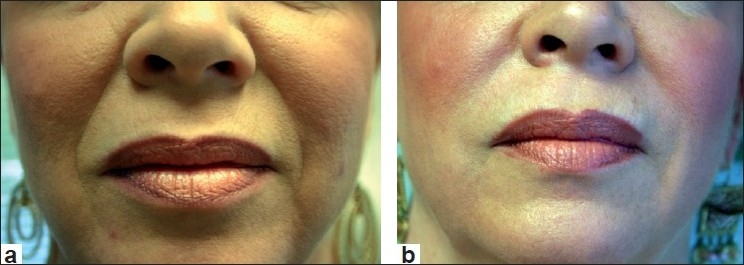
Before treatment (a) and after treatment with Restylane (1.0 cc) to nasolabial folds (b)

**Figure 2:**
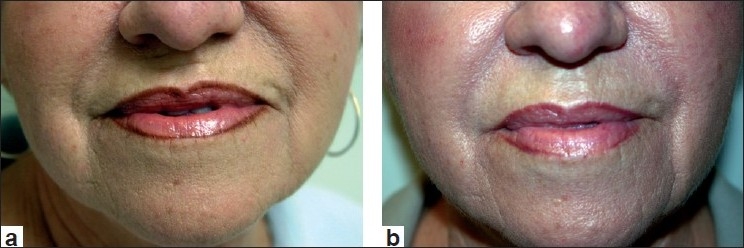
Before treatment (a) and post treatment with Restylane (1.0 cc) to the nasolabial folds and marionettes lines (b)

Following the approval of Restylane, Perlane received its FDA approval for deeper dermal defects, especially for those have deep nasolabial folds and for other lines and wrinkles that require a larger particle size HA filler. Lastly, clinical studies are at the concluding stage at this time, on a new Restylane product with lidocaine incorporated into the syringe itself. More information on this product should be available soon. Clinical examples of Restylane are shown in Figures 1 and 2.

b) Juvederm family: The next group of HA is known collectively as Juvederm. Juvederm is manufactured by Lea Derm, a subsidiary of the Corneal Group (Paris, France). It was brought to the US by Inamed and was purchased several years ago by Allergan, Inc. Allergan, the makers of Botox™, are the current distribution source worldwide for Juvederm. There are two current formulations of Juvederm available in the US – Juvederm Ultra and Juvederm Ultra Plus. Six different formulations of Juvederm have been developed by Corneal, with differing concentrations of HA in each formulation, ranging from 18 mg/ml to 30 mg/ml. Both the available US products contain 24 mg/ml of HA, respectively, with Juvederm Ultra Plus containing 24 mg/ml of HA in high viscosity. Both the US Juvederm formulations were FDA approved in June 2006 – Juvederm Ultra for deep wrinkles and defects and Juvederm Ultra Plus for deeper furrows, such as, the nasolabial folds. The Juvederm family is produced from the bacterial fermentation of equine streptococci. The HA is cross-linked with a patented single-phase BDDE-phosphate buffered from 6.5 – 7.3 pH. With a higher concentration of HA and more cross-linking than other HA fillers, it has been suggested that the Juvederm family of products may persist longer than other HA fillers, and also have a smoother injection flow.[20]

Baumann L *et al.*,[21], in an important clinical trial, compared three Juvederm products, with Zyplast collagen, in the treatment of nasolabial folds. Four hundred and twenty-three patients completed the clinical trial of a 24-week evaluation. Over 300 patients received an additional treatment, at the conclusion of the clinical trial in order to further examine the long-term efficacies of these products. Results from this multicenter clinical evaluation showed that both, the Juvederm family, and the Zyplast collagen, showed significant improvements at all points during the course of the 24 week clinical trial. The three products of the Juvederm family studied showed a significantly greater efficacy than the bovine collagen product; the efficacy increased with time and was greatest at 24 weeks after the last treatment. Utilizing a four-point scale, an improvement of at least one point was seen in more than 80% of the Juvederm-treated patients compared to a 0.5 improvement, on an average, in the Zyplast-treated patients. At the end of 24 weeks of injection, long-term results showed that there was 57% improvement at eight months, 37% at 10 months, and 18% at 12 months.

Adverse events were similar for both the Juvederm side and the Zyplast side that were treated, and were similar for all of the Juvederm products studied. Mild-to-moderate treatment site reactions were seen in a majority of patients, all of which resolved within seven days. No long-term adverse reactions were noted. Patient preference data suggested a 78% preference for Juvederm 30, 88% for Juvederm 24HV, and 84% for Juvederm 30HV. From this clinical study, Juvederm 24HV and Juvederm 30HV were chosen for the US market, both of which contained 24 mg/ml of HA, with Juvederm Ultra having 9% cross-linking, while Juvederm Ultra Plus had 11% cross-linking. Because of the long term follow-up during this multi-center clinical trial, the investigators were able to show longevity at one year following optimal correction, and therefore, were able to receive FDA clearance for up to one year.[22]

**Figure 3:**
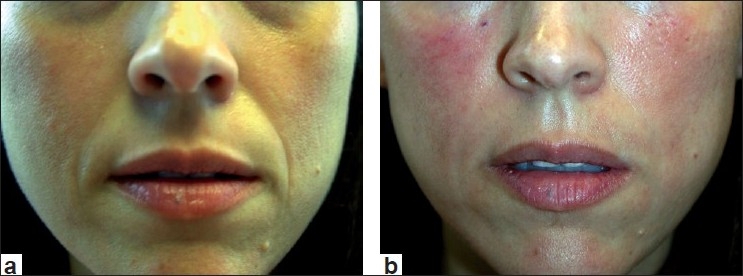
Before treatment (a) and immediately post treatment with Juvederm (1.0 cc) to the nasolabial folds and tear trough (b)

**Figure 4:**
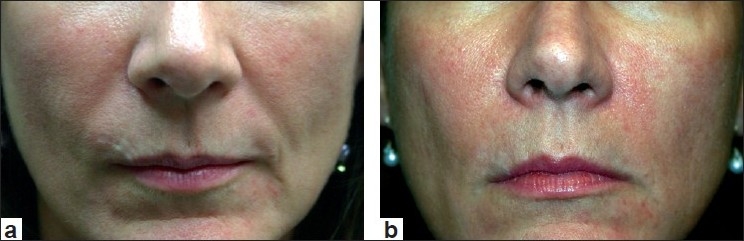
Before treatment (a) and immediately post treatment with Juvederm (1.0 cc) to the nasolabial folds and marionettes lines (b)

Most clinicians who utilize Juvederm have noted that it does inject easily through the syringe and that results are commonly observed for 6 to 12 months. Local injection site reactions are rare and there has been some discussion that the injection of Juvederm results in a more natural appearance than the other HA fillers, although no clinical studies with regard to this debate have been performed. Clinical examples of Juvederm are seen in Figures 3 and 4. A newer form of Juvederm, with lidocaine incorporated into the syringe itself, is now available in Europe, and pending FDA approval in the US at present.

c) Hydrelle: The next HA filler that received FDA approval was originally known as Elevess and is currently known as Hydrelle. Hydrelle is marketed through Coapt Systems (Palo Alto, CA, USA) and is manufactured by Anika Therapeutics (Bedford, MA, USA). Hydrelle contains the highest concentration of HA of all products, in the market at this time, 28 mg/ml, and it also contains 0.3% lidocaine, the first of the US products receiving FDA clearance for an HA with lidocaine. It is cross-linked with p-phenylene bisethyl carbodiimide or biscarbodiimide or BCDI, which is a novel HA cross-linker. Its source of HA is from equine streptococci. The US clinical pivotal study for Hydrelle (Elevess) studied 191 individuals who received Elevess in one nasolabial fold and CosmoPlast in the other nasolabial fold. Patients had significant improvement in the Elevess side at both four and six months following optimal correction. AEs were similar in both groups and not significant. They consisted mainly of treatment site reactions and resolved in the majority of cases within seven days.[23] Patients who still had improvement at the six-month time frame were eligible to enter a nine- (n = 90) and 12 month (n = 84) extension follow-up clinical trial. The patients maintained their improvement at these time frames as well; with the Elevess side showing more improvement than the CosmoPlast side.[24]

In clinical practice, this material is easy to inject with a 27 gauge needle; however, the 30 gauge needle supplied with the syringe makes the injection process a little more difficult than most of the other HA fillers. The addition of lidocaine is a benefit and most patients note a decrease in pain, almost immediately after the first injection into the skin.

d) Prevelle: The next HA filler that received FDA approval is known as Prevelle™ Silk. This product is the next generation of an earlier HA filler, known as Captique™, which is not available anymore. Captique was manufactured by the Genzyme Corporation (Cambridge, MA, USA) and was originally marketed by Inamed and then Allergan, and was later sold to the Mentor Corporation (Santa Barbara, CA, USA), who now markets the newer formulation of Captique, known as Prevelle Silk. more recently, Mentor HAS BEEN purchased by Johnson (Skillman, NJ USA). The product contains 4.5 – 6.0 mg/ml of HA, is 20% cross-linked with divinyl sulphone and has a gel particle size of 500 μm. On account of the low concentration of HA in the product, the clinical results were of short duration, in the three to six month time period.

Prevelle Silk combines Captique with 0.3% lidocaine and the pivotal trials for this product, conducted by Monheit *et al.*,[25] showed that Prevelle Silk had a significant difference in pain associated with the injection process and postprocedure pain. The majority of patients receiving Prevelle Silk do not have significant post-treatment erythema or post-treatment swelling. Patient preference was also significantly more in favor of the Prevelle Silk over Captique. Prevelle Silk is generally preferred for patients needing instant correction with very little potential for adverse effects. Genzyme and Mentor have more fillers on the horizon, which will be part of the Prevelle family. The first of these products should be available towards the end of 2009 or early 2010.

## PRODUCTS USED FOR SOFT TISSUE AUGMENTATION

a) Collagen: Is there still a role for a collagen product to be useful in today's world for soft tissue augmentation? The answer is definitely yes. A new ‘porcine’ collagen has become available for use in the US recently and is gaining market share, as dermatologists become more attuned to its injection techniques and its longevity once implanted. It is known commercially as Evolence and is manufactured by ColBar LifeScience Ltd. (Hertzelia, Israel), which is now part of Johnson and Johnson (OrthoNeutrogena Aesthetics, Skillman, NJ, USA). Evolence utilizes a specialized process of stabilization known as ‘glymatrix,’ in which there is ploymerization of the monomeric porcine collagen by glycation with ribose, a naturally occurring sugar. This glymatrix technology helps the porcine collagen form a three-dimensional gel network, which helps create a more stable, longer lasting filling material. The porcine source is from a closed herd in Australia and the glymatrix process takes place in Israel. Evolence received its FDA clearance in June 2008.

Clinical studies with Evolence have shown its effectiveness. Skin testing data by Shoshani *et al.*,[26] have demonstrated that there is virtually no immunogenicity to this product. Five hundred and thirty patients were evaluated for hypersensitivity reactions to Evolence and none were found in this clinical trial. Thus, skin testing for Evolence is not required. The first EU study on Evolence[27] evaluated Evolence in one nasolabial fold and Zyplast in the other nasolabial fold. None of the patients demonstrated skin test reactions and longevity of the Evolence was maintained upwards of 18 months following implantation. The US pivotal study by Narins *et al.*,[28] evaluated Evolence in one nasolabial fold and NASHA in the other nasolabial fold. A total of 149 patients were enrolled in this multicenter clinical trial. There were significant improvements noted in both nasolabial folds at six months (non-inferiority study), with more AEs noted on the NASHA side as compared to the Evolence side. An extension study for evaluation of patients receiving Evolence at 12 months was also performed and did show significant improvement in 12 months. The FDA approved a 12 month labelling for Evolence in June 2009. Further evaluations in a multicenter evaluation with Evolence continue at this time, especially in patients with coloured skin, with results expected to be available by the end of 2009.

**Figure 5:**
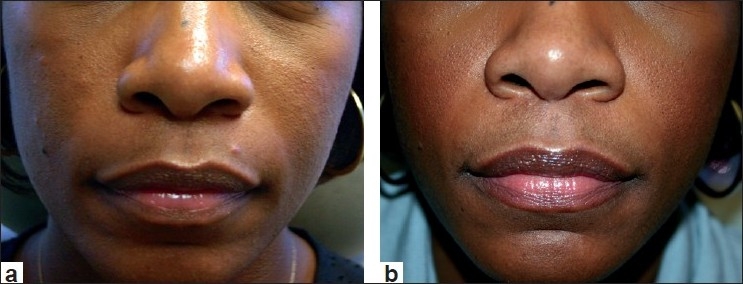
Before treatment (a) and after treatment with Evolence (1.0 cc) to left nasolabial fold (b)

**Figure 6:**
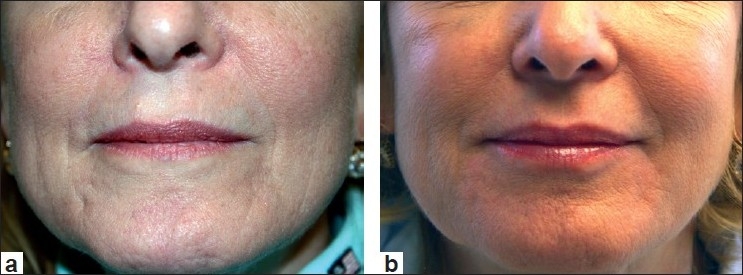
Before treatment (a) and after treatment with Evolence (1.0 cc) to marionette lines, upper lip, vertical lines (b)

A second Evolence, known as Evolence Breeze, is indicated for more superficial lines, wrinkles and lips. This product is available in many countries around the world; US clinical trials are supposed to start shortly. This implant requires a more sophisticated injection technique than that of the typical HA filler. On account of its unique nature and processing, Evolence needs approximately one hour to ‘set in’. Therefore, massaging immediately after the injection itself allows for proper moulding of the implant, which will then be in place for the duration of the implant. Some patients continue to feel the effect of the injection at the treatment site for several days to weeks after being injected with Evolence, but this resolves and patients enjoy this implant for many months. In fact, most patients note that this product lasts for 12 to 18 months. Clinical examples of Evolence are shown in Figures 5 and 6.

b) Radiesse: Radiesse, a semipermanent filler also known as calcium hydroxylapetite (CaHA), contains synthetic CaHA microspheres (30%) suspended in a carboxy-methylcellulose resorbable aqueous gel carrier (70%). This process allows for the body's stimulation of collagen. Skin testing is not required for Radiesse injections. Radiesse was approved by the FDA in December 2006, and is indicated for the treatment of facial wrinkles and folds, as well as, the correction of facial wasting as a result of HIV-associated lipoatrophy. It was the first filler to receive these two FDA indications. Pivotal US clinical trials for both these indications showed significant improvements[29,30] and many studies have demonstrated longevity with Radiesse, for over one year and up to two years.[31–33]

Radiesse has found a niche role, with many clinicians who are looking for a more ‘robust’ filler and long-lasting results. It also has become one of the favourite fillers for hand rejuvenation, utilizing injections of Radiesse into the dorsum of the hands and then massaging to mould the Radiesse into the skin. Many clinicians have also incorporated lidocaine into the Radiesse syringe through an adaptor process – this has recently received FDA approval as it has become the standard of care.

c) Sculptra: Sculptra, or poly-L-lactic acid, another semipermanent filler, has been available in the US market for the past several years with FDA approval in 2004, to treat HIV-associated lipoatrophy. In July 2009, Sculptra received FDA clearance from the FDA to treat lines and wrinkles for aesthetic considerations. It is best used as a volume enhancement treatment and requires several treatment sessions to achieve the desired effect. The early Sculptra studies in Europe[34,35] showed the efficacy of this product. The first of these, known as the VEGA study[34], evaluated 50 individuals and found an increase in skin thickness, which was significant, in all the studies conducted at various times. The material was found to be persistent after a full correction for upwards of two years. Visual improvements, confirmed by serial photographic analysis, confirmed the results. The second European study[35], known as the Chelsea and Westminster Study, evaluated 29 patients. Once again, an increase in skin thickness was found in all the patients studied. There was a mean change of 4 to 6 mm noted at 12 weeks following correction. Also, improvements in anxiety and depression scores were noted in these subjects, as a result of increased self-esteem due to this therapy.

In the US, two pivotal FDA clinical trials were performed in HIV-associated lipoatrophy patients. They are known as the APEX002 (n = 95) and the Blue Pacific (n = 68) studies.[36,37] Both these studies showed the effectiveness of Sculptra in HIV-associated lipoatrophy.[36,37] Many other, recent studies confirm these original trials and the effectiveness of Sculptra for several years duration.[38,39]

As noted, for Sculptra to achieve its full correction, the patients need a series of injections. The injections are usually spaced four to six weeks apart. It is important to inform the patients with HIV-lipoatrophy about this fact- that two to four injection sessions may be required for the poly-L-lactic acid to stimulate new collagen and reverse the signs of lipoatrophy. However, for cosmetic enhancement, one to three sessions are usually sufficient. There are also various techniques to prepare the product for the injection and each clinician will develop his/her ‘favorite’ technique. The authors usually mix 5 cc of sterile water with 1 cc of 0.3% lidocaine and let the medicine set for 24 hours prior to the injection of the Sculptra. Most of the experience with Sculptra, in the US, has been with patients suffering from HIV associated lipoatrophy and there is no doubt that the product has changed their lives for the better.

d) Artefill: The last filler that is being discussed in this article is a permanent filler, known as ArteFill, being promoted by Suneva Medical Inc. (San Diego, CA, USA). This is an interesting filler, composed of polymethyl-methacrylate (PMMA) microspheres suspended in a rapidly dissolving bovine collagen carrier, with 0.3% lidocaine added to the syringe. It was designed in this fashion to induce ‘reactive’ long-term collagen deposition. The PMMA microspheres are from 30 to 50 μm in size, too big to be phagocytised within the body, but small enough to be easily injected through a 26 gauge needle. This product has had several previous lives, first as ArtePlast, and then ArteColl, and now ArteFill. The previous generation products differ from today's products in many ways and it is sufficient to say the current product is safe and effective. ArteFill received FDA approval in October, 2006. In the US pivotal clinical trial, ArteFill was compared to Zyplast or Zyderm collagen in the nasolabial folds.[40] Two hundred and fifty-one patients were enrolled in this trial and at six months, the collagen sides were crossed-over to receive ArteFill also. Furthermore, at six months, a significant change was noted in the nasolabial folds which received ArteFill, while the collagen sides had returned to their baseline. AEs were similar between both the groups.[40] Safety studies for ArteFill continued successfully for 12 months. Since the US pivotal clinical trial, there is an ongoing five-year safety trial for ArteFill, which is currently in year three. The purpose of this study is to examine the long-term effects of ArteFill, including its efficacy as well as its safety.

While ArteFill has had a checkered history in the US market, it is a very good filler for patients with deep dermal defects, who understand that the filler being placed will last anywhere from one to five years, depending on numerous factors, including the level of skill of the injector and the proper placement of the product.

## SUMMARY

Soft tissue augmentation remains a growing field. There are very good fillers currently available and many more on their way. We, as dermatologists, have an array of treatment options available to help rejuvenate the skin of our patients, and dermal fillers are part of that process, along with botulinum toxin A, lasers and light sources and appropriate skin care. Combining different modalities will yield the best results.

It is often hard to study and be familiar with ‘all’ the fillers. It is appropriate to understand one or two and use them well. One should be aware of what makes each filler unique and where each filler might have its optimal place for injection. Learning proper injection techniques is important and learning from your peers is an opportunity that will allow you to acquire the skills you need to make you the best injector possible.
